# Genome-Wide Analysis of Repeat Diversity across the Family Musaceae

**DOI:** 10.1371/journal.pone.0098918

**Published:** 2014-06-16

**Authors:** Petr Novák, Eva Hřibová, Pavel Neumann, Andrea Koblížková, Jaroslav Doležel, Jiří Macas

**Affiliations:** 1 Biology Centre ASCR, Institute of Plant Molecular Biology, České Budějovice, Czech Republic; 2 Institute of Experimental Botany, Centre of the Region Haná for Biotechnological and Agricultural Research, Olomouc, Czech Republic; Leibniz-Institute of Plant Genetics and Crop Plant Research (IPK), Germany

## Abstract

**Background:**

The banana family (Musaceae) includes genetically a diverse group of species and their diploid and polyploid hybrids that are widely cultivated in the tropics. In spite of their socio-economic importance, the knowledge of Musaceae genomes is basically limited to draft genome assemblies of two species, *Musa acuminata* and *M. balbisiana*. Here we aimed to complement this information by analyzing repetitive genome fractions of six species selected to represent various phylogenetic groups within the family.

**Results:**

Low-pass sequencing of *M. acuminata*, *M. ornata*, *M. textilis*, *M. beccarii*, *M. balbisiana*, and *Ensete gilletii* genomes was performed using a 454/Roche platform. Sequence reads were subjected to analysis of their overall intra- and inter-specific similarities and, all major repeat families were quantified using graph-based clustering. Maximus/SIRE and Angela lineages of *Ty1/copia* long terminal repeat (LTR) retrotransposons and the chromovirus lineage of *Ty3/gypsy* elements were found to make up most of highly repetitive DNA in all species (14–34.5% of the genome). However, there were quantitative differences and sequence variations detected for classified repeat families as well as for the bulk of total repetitive DNA. These differences were most pronounced between species from different taxonomic sections of the Musaceae family, whereas pairs of closely related species (*M. acuminata*/*M. ornata* and *M. beccarii*/*M. textilis*) shared similar populations of repetitive elements.

**Conclusions:**

This study provided the first insight into the composition and sequence variation of repetitive parts of Musaceae genomes. It allowed identification of repetitive sequences specific for a single species or a group of species that can be utilized as molecular markers in breeding programs and generated computational resources that will be instrumental in repeat masking and annotation in future genome assembly projects.

## Introduction

Bananas are giant perennial herbs belonging to the genus *Musa*, which are grown in tropical and subtropical regions. Edible sweet and starchy banana cultivars provide a staple food for many millions of people and are a major export commodity. Based on a set of morphological descriptors and basic chromosome number, the genus *Musa* has been traditionally subdivided into four sections: *Eumus*a (x = 11), *Rhodochlamys* (x = 11), *Australimusa* (x = 10), and *Callimusa* (x = 9 or 10) [1]. However, this classification has been often questioned. The recent use of a variety of molecular markers provided detailed information on *Musa* genetic diversity and phylogenesis [2]–[8]. Finally, in 2013, the *Australimusa* and *Callimusa* sections were merged into the section *Callimusa*, and sections *Eumusa* and *Rhodochlamys* were merged into the section *Musa* [9].

Most of the edible bananas are vegetatively propagated diploid and polyploid forms of *M. acuminata* (A genome, 2n = 2x = 22) and hybrids that originated from crosses between *M. acuminata* and *M. balbisiana* (B genome, 2n = 2x = 22) [10] belonging to the section *Musa*. Although there is some inconsistency in the classification of cultivated banana clones, it has been estimated that there are at least 1000 different cultivars grown worldwide [11]. Another group of edible cultivars, called Fei, represents a separate line of banana evolution and comprises a different species complex classified in the section *Callimusa*. The section is represented by a number of wild *Musa* species, including *M. maclayi*, *M. peekelii*, and *M. lolodensis*, the most probable progenitors of edible Fei bananas [12]–[15] and *M. textilis* (T genome). Fei bananas are parthenocarpic and vegetatively propagated like other edible banana clones. However, they were domesticated independently from the cultivars of the section *Musa*.

The production of bananas has been seriously threatened by the increasing range of fungal, viral, and insect diseases. At the same time, breeding of improved cultivars is hampered by seed sterility, unknown origin of the cultivated clones, and poor knowledge on genetic diversity of the genus *Musa*. The nuclear genome of *Musa* species is relatively small (1C∼600 Mbp; [16], [17]), and previous studies showed that ∼55% of the genome is represented by repetitive DNA, especially different types of retroelements [18]–[20]. Genomic repeats evolve more rapidly than coding sequences, and plant geneticists and breeders found them a unique source of molecular markers to map important genes, analyze genetic diversity, and study processes of speciation and genome evolution [21]–[23].

Global characterization of complex populations of plant genomic repeats recently has been made feasible by combining next-generation sequencing technologies with newly developed bioinformatic tools [24], [25]. This approach also led to the first characterization of major repeat types in the genome of ‘Calcutta’, a clone of *M. acuminata* ssp. *burmannica*, a popular male parent in a number of breeding programs [19]. Additional information about repeat composition and organization in this species (ssp. *malaccensis*) was obtained after producing a draft sequence of a double haploid individual derived from wild accession ‘Pahang’ [20]. Finally, initial data about repeats in another species, *M. balbisiana* (variety ‘Pisang Klutuk Wulung’) recently have been released along with its draft genome [26]. Apart from *M. acuminata* and *M. balbisiana* from the section *Musa*, similar information is missing for the section Callimusa and other representatives of the Musaceae family.

In this study, we employed bioinformatic analysis of low-pass genome sequencing data to get deep insight into repeat composition of Musaceae genomes. We selected five *Musa* species and one representative of the genus *Ensete* for comparative analysis of repetitive fractions of their genomes in order to (1) identify and quantify major groups of repetitive sequences, (2) assess sequence diversity of repeats between the species and investigate its correlation with the phylogeny of the Musaceae family, and (3) generate bioinformatic resources for development of repeat-based genome-specific markers and for repeat identification and annotation for future genome assembly projects.

## Results

### Low-pass genome sequencing, estimation of repeat proportions, and similarities between the species

**Figure 1:**
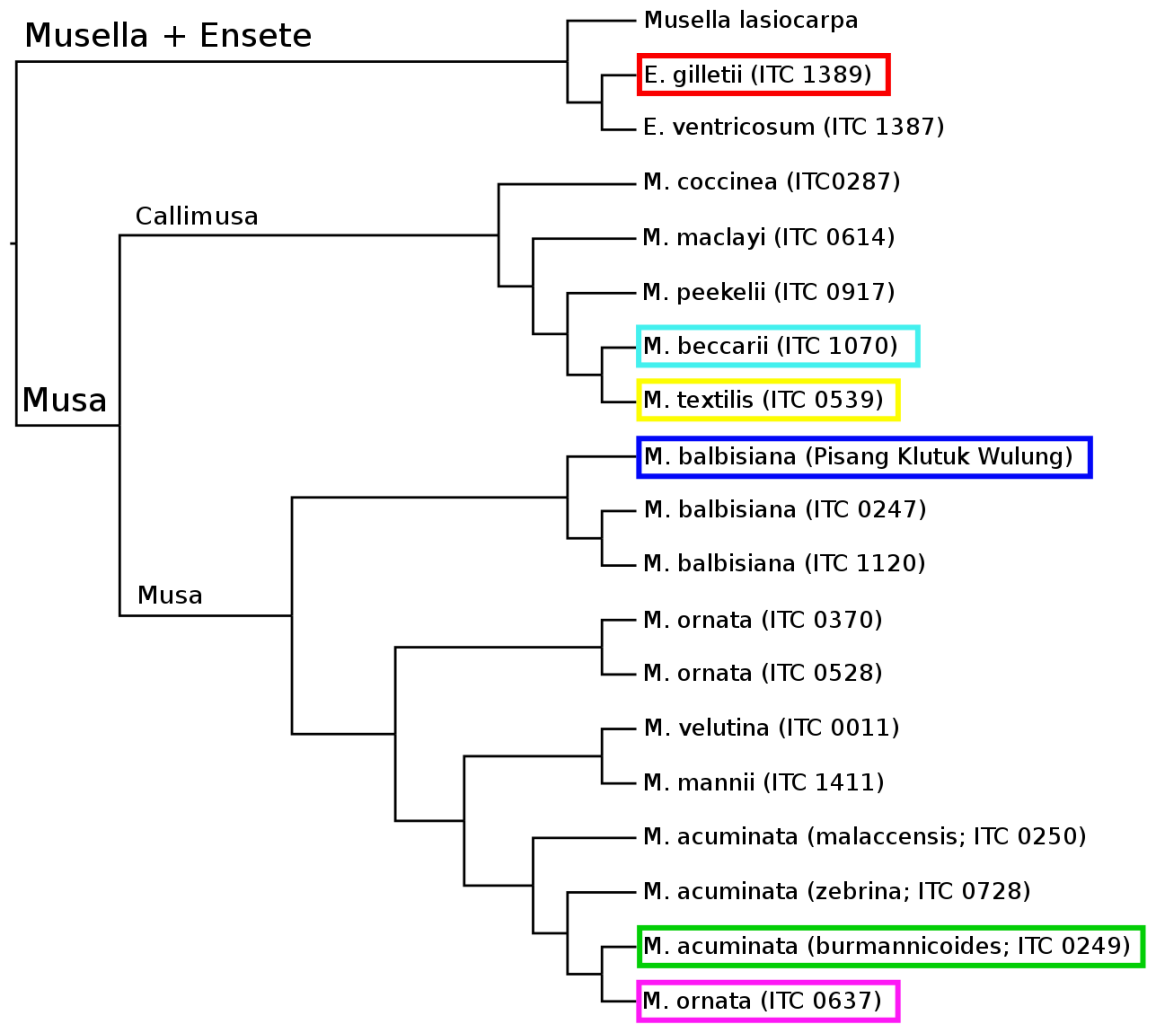
Evolutionary relationship between species of Musaceae family. Phylogeny estimated from ITS data using BioNJ. Six genomes selected for repeat analysis are highlighted.

Five representatives of the genus *Musa* and one *Ensete* species were selected for analysis to cover various phylogenetic clades of the Musaceae family (Fig. 1). They possess relatively small genomes with only moderate differences between the species, ranging from 567 to 763 Mbp/1C [16], [17], [27]. Whole-genome shotgun sequencing was performed using Roche/454 technology, and resulting reads were trimmed to the same length of 200 nucleotides. The same amount of reads (380,599) from each species was used for analysis, providing 0.10–0.13× genome coverage (Table 1). For this coverage, the probability of detecting repetitive sequences with 10 and 100 copies per haploid genome was 63–74% and >99.9%, respectively [28].

**Table 1:** Sequenced species.

genus	section	species	Genome	genome size [Mb]	proportion of genome analyzed [%]	code	ITC	Number of chromosomes
Musa	Musa	M.acuminata	AA	599	12.7	MACU	0728	2n = 2x = 22
	Musa	M.ornata		635	12.0	MORN	0637	2n = 2x = 22
Musa	Musa	M.balbisiana	BB	567	13.4	MBAL	1120	2n = 2x = 22
	Callimusa	M.beccarii		763	10.0	MBEC	1070	2n = 2x = 18
	Callimusa	M.textilis	T	701	10.9	MTEX	0539	2n = 2x = 20
Ensete		E.gilletii		591	12.9	EGIL	1389	2n = 2x = 18

**Figure 2:**
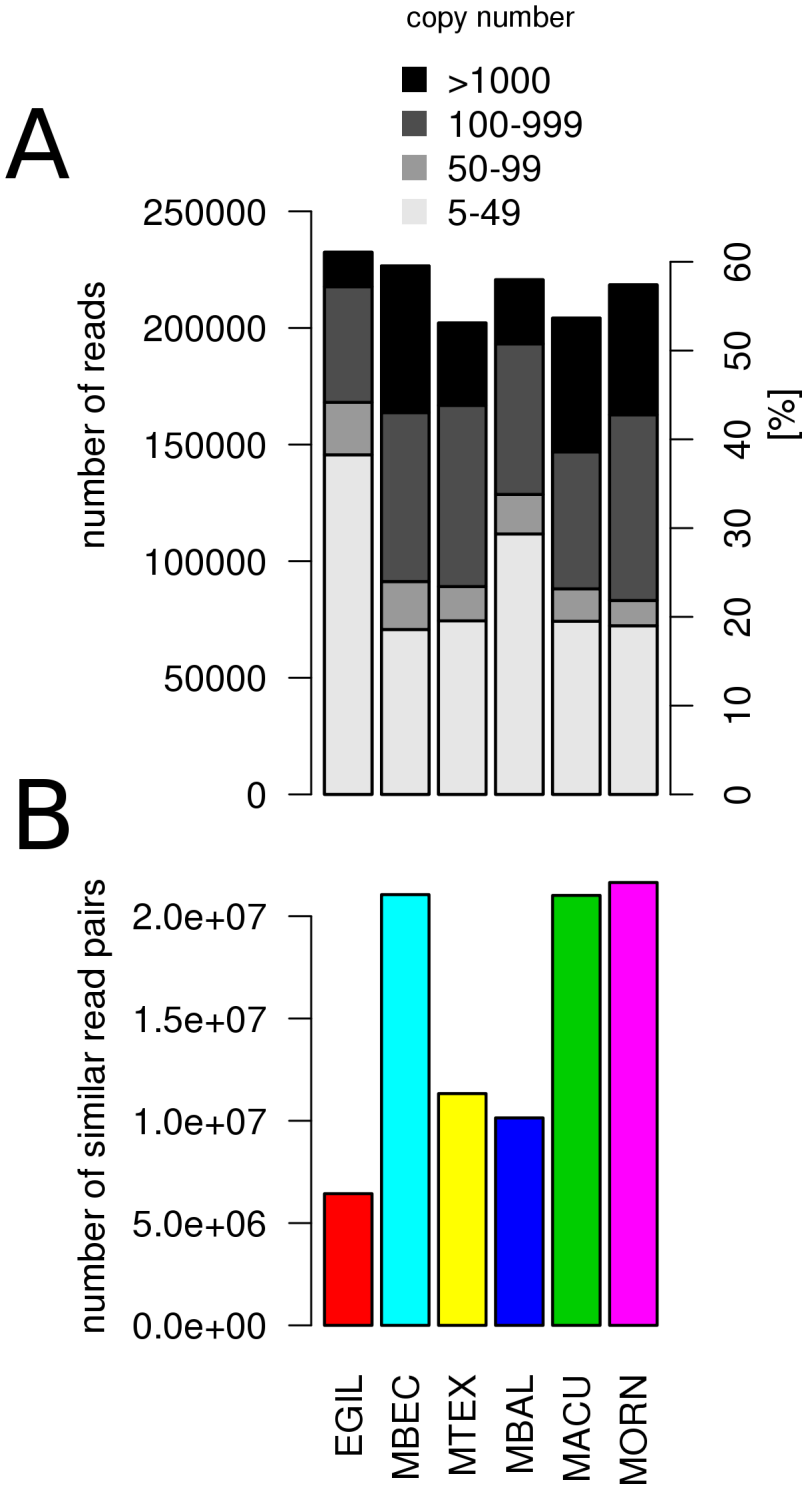
All-to-all similarity comparison of sequence reads from six Musaceae species. (A) The barplot shows the total number of reads with detected similarity hits which is proportional to size of repetitive fraction of the genome. As the number of similarity hits to each read is also proportional to its copy number, reads derived from repetitive elements can be divided into low, medium and high copy number fractions. (B) The total number of similarity hits that correspond to number of read pairs with similarity.

Sequence reads derived from genomic repeats were identified and quantified based on the number of similarity hits generated in all-to-all read comparisons. In principle, low sequencing coverage and similarity threshold used in this analysis (90% identity over 55% of the sequence length) provide a small chance of detecting hits between single-copy genomic sequences. Thus, most of the similarities are expected between the reads representing repetitive sequences and, their frequencies are proportional to copy number of corresponding repetitive elements in the genome. In the first part of the analysis, similarity hits were investigated separately for reads from each species compared to themselves, thus providing information about repeat proportions in individual genomes. There were similar amounts (55–60%) of reads generating at least one similarity hit in all species. However, there were differences in proportions of moderately (>100 copies/1C) and especially of high-copy (>1000 copies/1C) repeats that were most abundant in *M. beccarii*, *M. acuminata*, and *M. ornata* (Fig. 2A). Higher proportions of high-copy repeats in these species were also evident from differences in total numbers of similarity hits (Fig. 2B).

**Figure 3:**
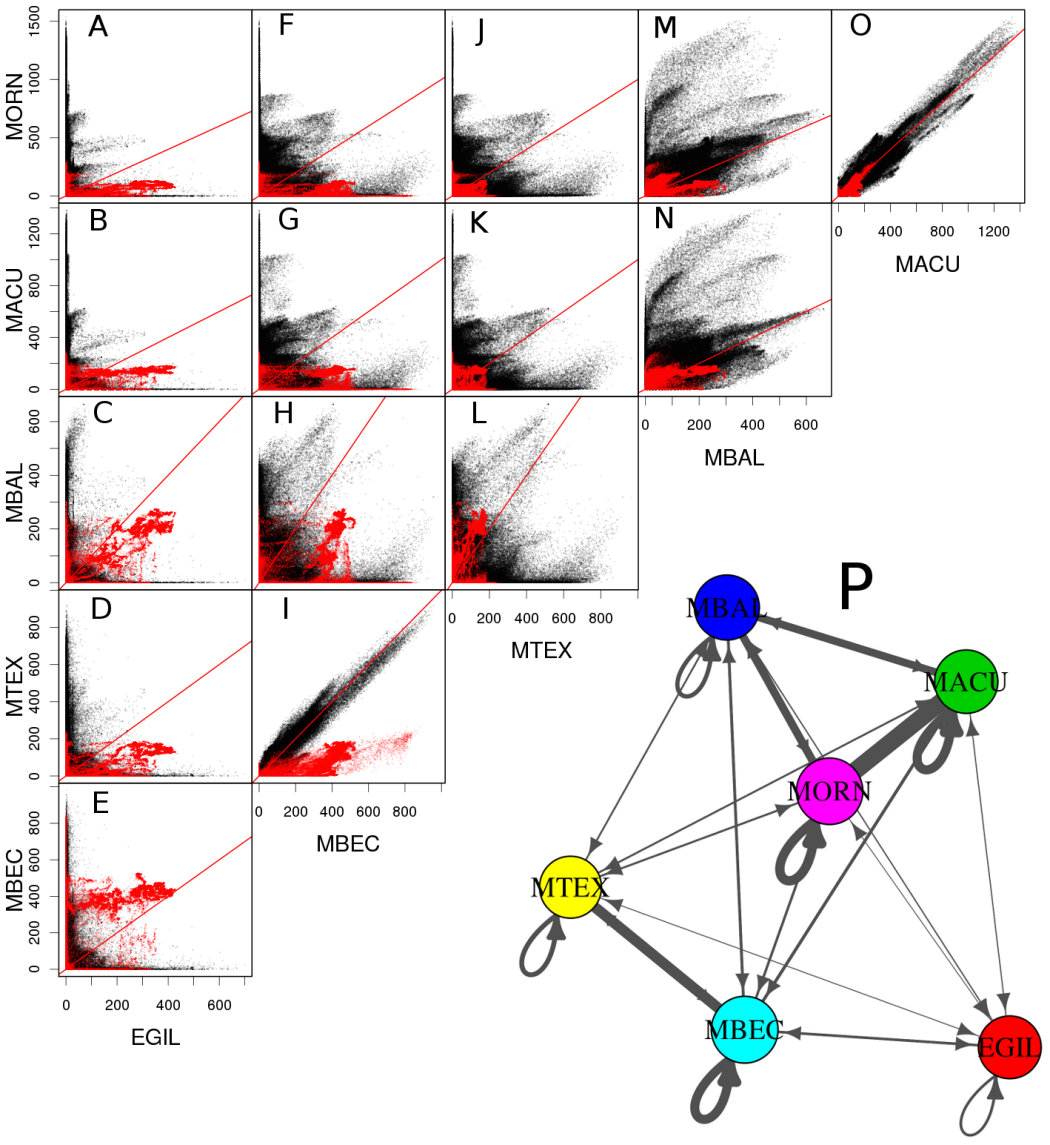
Comparison of genomic abundance of analyzed reads in all six species. (A–O) Scatter plots show pairwise comparisons of all analyzed sequences between pairs of species. Each spot corresponds to one sequence read. For each sequence read, the number of similarity hits in each species is displayed (this number is proportional to genomic representation of a particular sequence). Red diagonal line marks the position of sequences with equiproportional genomic representations. Sequences with differential genomic representation between species deviate from diagonal. The 45S rDNA sequences are shown in red. (P) Graph summarizing the number of identified read similarities between and within genomes. Width of the lines connecting nodes of the graph correspond to the number of identified similarity hits between sequence reads from different species (straight lines) and within the same species (loops).

To evaluate overall differences in sequence composition and abundance between pairs of species, inter-specific comparisons of read similarities were performed and visualized in a form of scatter plots, where dots represented reads and their positions were determined by numbers of similarity hits in both species (Fig. 3). This analysis revealed remarkable similarity of repeat composition of *M. acuminata* and *M. ornata* genomes, resulting in a diagonal pattern of the dot positions, which was due to similar abundance of corresponding sequences in both genomes (Fig. 3O). High similarity was also revealed between repeats from *M. beccarii* and *M. textilis*, except for much higher genomic proportion of 45S rDNA repeats in *M. beccarii* (Fig. 3I). Quantification of rDNA proportions revealed that its differential amplification accounts for most of the genome size difference between these species. The detected quantitative differences in 45S rDNA content are also in agreement with previously published FISH experiments which have shown higher number of 45S rDNA sites in *M. beccarii* and *E. gilletii* [17]. On the other hand, repeat composition of *E. gilletii* was the most divergent from other species (Fig. 3A–E). The diagram in Fig. 3P summarizing all similarities demonstrates this divergence of *E. gilletii* from all other species and also shows a closer relationship of *M. balbisiana* to the *M. acuminata*/*M. ornata* group.

### Classification and comparative analysis of major groups of repeats

To classify repetitive sequences and identify their orthologous groups present in individual genomes, the identified intra- and inter-specific similarities of sequence reads were analyzed using the RepeatExplorer pipeline [25]. The pipeline runs graph-based clustering algorithm [24] to identify groups of frequently overlapping reads representing families of repetitive elements followed by similarity- and structure-based repeat identification tools that aid in repeat classification. Classification of repeats in the 106 largest clusters exceeding in size 0.03% of the analyzed reads revealed that *Ty1/copia*, *Ty3/gypsy*, and rDNA repeats make up the majority of highly and moderately repeated sequences in the Musaceae genomes (Table 2, Fig. S1). *Ty1/copia* LTR-retrotransposons, mainly represented by Maximus and Angela lineages, were 2–4-fold more abundant than *Ty3/gypsy*. However, proportions of most repeat groups varied between the species even for the most abundant elements like *Ty1/copia* Maximus, which was much less abundant in *E. gilletii* and showed almost 1.5-fold variation in abundance between *Musa* species. Depending on the genome size and repeat content, the annotated repeats corresponded to 24% (*E. gilletii*) up to 44% (*M. beccarii*) of the genome. The rest of the repeats including mainly low-copy sequences forming small repeat clusters made up 26–43% (Table 2). In summary, repetitive sequences occupy about 66–71% in all genomes.

**Table 2:** Genome proportions of repetitive sequences.

					Genome							
Repeat		Lineage/class	Clade	alternative names	EGIL	MBEC	MTEX	MBAL	MACU	MORN	DH pahang assembly*	PKW assembly*
**LTR retroelements**	**Ty1/Copia**	Maximus-SIRE			4.277	14.860	17.744	16.388	19.483	21.794		12.26
		Angela			5.765	8.354	8.797	5.088	4.471	4.800		4.81
		Tork		Tnt	1.313	1.513	1.517	.943	1.380	1.267		1.07
		Ale		Hopscotch	.388	.728	.571	.358	.363	.409		
		Ivana			.989	.315	.332	.191	.220	.230		
		TAR		Tont	.013	.040	.054	.049	.061	.072		
		**Total Ty1/Copia**			**12.745**	**25.809**	**29.015**	**23.016**	**25.977**	**28.573**	**21.55**	
	**Ty3/Gypsy**	Chromoviruses	Reina		3.057	4.081	3.935	3.022	5.189	4.612		2.57
			Tekay		.437	2.365	1.685	1.763	2.427	2.714		1.45
			Galadriel	Monkey	1.135	1.365	1.020	.585	.641	.536		0.68
			CRM		.358	.949	.877	.589	.567	.626		0.25
			unclassified		.262	.050	.000	.000	.000	.000		
		Tat			1.693	.260	.290	.010	.060	.047		
		**Total Ty3/Gypsy**			**6.942**	**9.071**	**7.808**	**5.970**	**8.885**	**8.535**	**9.65**	
**Other**												
	**pararetrovirus**				.000	.000	.000	.010	.023	.057		
	**LINE**				.490	.790	.472	.492	1.214	1.120	5.41	0.39
	**DNA transposon**				.118	.281	.272	.269	.253	.224	1.24	0.17
	**Tandem Repeats**	rDNA			3.726	7.736	2.067	2.459	2.994	2.239		
		Satellites			.005	.443	.122	.592	.247	.330		0.13
**Annotated repetitive total**					**24.025**	**44.129**	**39.756**	**32.808**	**39.592**	**41.078**		
**Unclassified repetitive**					43.084	26.742	25.804	35.958	27.897	28.737		
**All repetitive total**					**67.109**	**70.870**	**65.560**	**68.766**	**67.489**	**69.815**		26.85
**unknown low copy**					32.891	29.130	34.440	31.234	32.511	30.185		

*Repeat proportion estimates from [20], [26].

**Figure 4:**
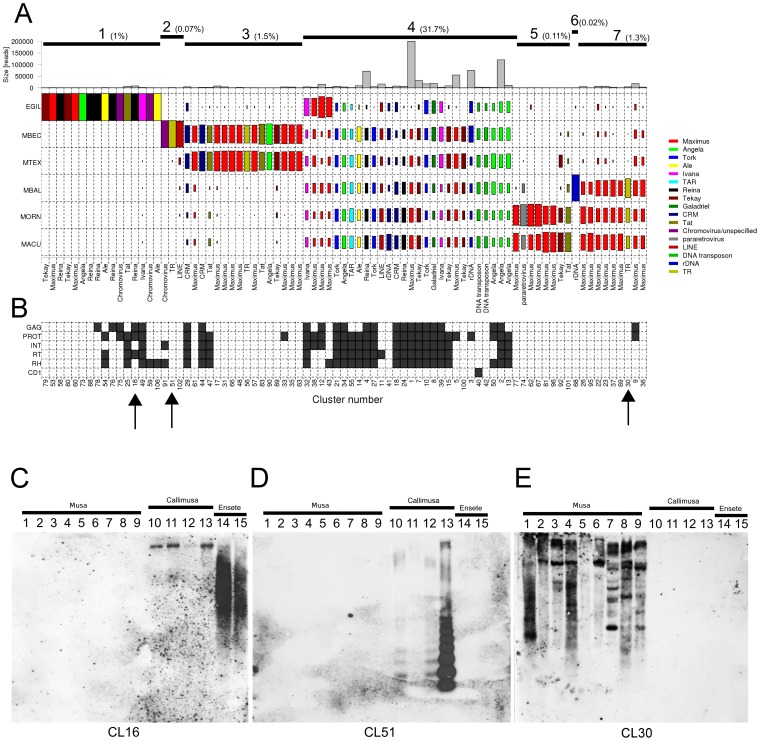
Comparative analysis of Musaceae species based on the cluster composition. (A) Sequence composition of the largest clusters is shown. The size of the rectangle is proportional to the number of reads in a cluster for each species. Bar plot in the top row shows the size of the clusters as number of reads. Color of the rectangles correspond to the type of the repeat. Upper lines label groups of clusters discussed in the text. The percentage of reads included in the group is shown in parentheses. (B) The presence of mobile element protein domains in the contig assembled from sequences within the cluster. Only clusters that were annotated are shown. (C–E) Validation of clustering results by Southern blot. Genomic DNA from 15 species was probed with sequences derived from clusters CL16, CL51. and CL30. The lanes contain DNA from 1/*M. acuminata* ssp. *zebrina* (ITC 0728), 2/*M. acuminata* ssp. *malaccensis* (ITC 0250), 3/*M. acuminata* ssp. *burmannicoides* (ITC 0249), 4/*M. ornata* (ITC 0637), 5/*M. mannii* (ITC 1411), 6/*M. ornata* (ITC 0528), 7/*M. balbisiana* (ITC 1120), 8/*M. balbisiana* (‘Pisang Klutuk Wulung’), 9/*M. balbisiana* (ITC 0247), 10/*M. peekelii* (ITC 0917), 11/*M. maclayi* (ITC 0614), 12/*M. textilis* (ITC 0539), 13/*M. beccarii* (ITC 1070), 14/*E. ventricosum* (ITC 1387), and 15/*E. gilletii* (ITC 1389).

An inherent feature of the clustering analysis when applied to sequence data from multiple species is that orthologous repeat families from different species are grouped to the same clusters. This facilitates identification and quantification of repeats that are shared between the species as well as detection of species-specific sequences. The analysis revealed that a large part of the repeat clusters representing various families of LTR-retrotransposons, DNA transposons, long interspersed nuclear elements (LINE), and rDNA was shared by all *Musa* species and, to a smaller extent, also with *E. gilletii* (Fig. 4A, group 4). This group of clusters was also the most significant in terms of genome proportions, containing about 32% of analyzed reads (89% of annotated repeats). Smaller groups 3, 5, and 7 included clusters that were shared by two or three species. They comprised mostly non-coding parts of LTR-retrotransposons (e.g., LTR sequences), which evolve more rapidly than their gag-pol regions. Groups of species sharing these sequences included *M. beccarii*/*M. textilis* (group 3), *M. acuminata*/*M. ornata* (group 5), and *M. acuminata*/*M. ornata*/*M. balbisiana* (group 7), which is in agreement with overall read similarities between the species presented in Fig. 3. The largest number of species-specific clusters, including complete retrotransposon sequences, was detected for *E. gilletii*, which was in agreement with the phylogenetic divergence of this species.

To verify some of the differences in repeat composition revealed by the clustering analysis, three putative section or species-specific repeats were detected in a set of Musaceae species using Southern blot hybridization (Fig. 4C–E). Experimental results were in all cases in agreement with the output of bioinformatic analysis. The probe derived from CL16, classified as a Reina lineage of *Ty3/gypsy* elements specific for the genome of *E. gilletii*, produced strong hybridization signals in species of the *Ensete* genus, with weak or no labeling of genomic DNAs of *Musa* species (Fig. 4C). An *M. beccarii*-specific tandem repeat found in the cluster CL51 also showed predominantly species-specific hybridization pattern with only minor signals in related species from *Callimusa* sections (Fig. 4D). The probe derived from tandem-like repeat CL30 present in sequence reads from *M. acuminata*, *M. ornata*, and *M. balbisiana* was confirmed to be specific for section Musa, which includes these three species (Fig. 4E).

**Figure 5:**
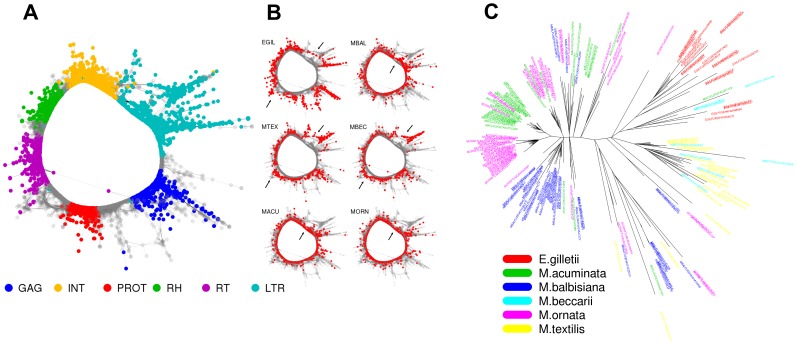
Variability of sequences within cluster CL18. (A) Sequence reads are represented by nodes of the graph and reads with identity of at least 90% with minimal overlap of 110 nt are connected by lines. Graph layout was calculated using the 3D version of the Fruchterman and Reingold algorithm [55] from which a 2D projection is shown. Reads are colored based on their similarity to conserved coding domains of LTR retrotransposons. Reads from LTR regions are colored by light blue. (B) Nodes of the graph are colored based on their species of origin. The six identical graphs show reads derived from each species in red; remaining reads are gray to highlight species-specific parts of the graphs. The parts of the graphs that represent the most variable sequence regions in CRM CL18 element, which can differentiate between species, are labeled by black arrows. (C) Maximum-likelihood tree based on nucleotide alignment of sequences covering the reverse transcriptase protein domain of CRM CL18. Sequence read names are colored based on the species of origin.

In addition to identifying species- and section-specific repeats based on their presence in different clusters, it was possible to reveal more subtle sequence variations even for repeats grouped into the same cluster. An example of this variability is presented in Fig. 5A–B, showing a graph structure of the cluster CL18 representing a family of *Ty3/gypsy* elements belonging to the chromovirus CRM clade. In higher plants, transposition of this group of LTR-retrotransposons is targeted to centromeres, and this localization has also been reported for *M. acuminata* CRM elements [29]. The cluster graph is composed of nodes representing individual sequence reads and edges connecting reads with similarities exceeding the specified threshold [24]. Since the node distances are inversely proportional to read similarities, the graph layout reflects sequence variability of the element copies in the genome, and in the case of reads from different species, reveals its inter-specific variability. This variability (Fig. 5B) as well as phylogenetic analysis of reverse transcriptase (RT)-coding sequences extracted from the reads (Fig. 5C) were in agreement with phylogenetic reltionships of the species (Fig. 1). In addition, the RT-based phylogenetic tree provided better discrimination of sequences from closely related species and contained some species- or section-specific branches with shorter edges, implying recent amplification of CRM elements in *M. acuminata*, *M. ornata*, and *M. balbisiana*.

### Preparation of repeat databases and their use for repeat annotation in assembled genomes

**Figure 6:**
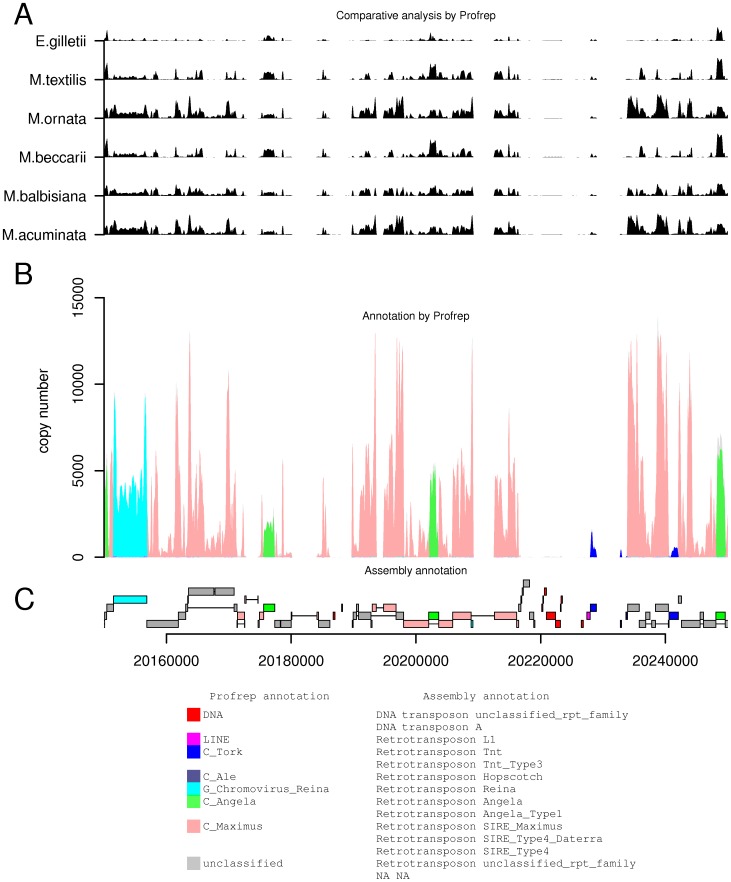
Analysis of 100-Pahang assembly using Profrep. Genomic sequence Chr9:20,150,000–20,250,000 together with repeat annotation was obtained from the Banana Genome Hub (http://banana-genome.cirad.fr/) and analyzed using the Profrep tool against our Musaceae repetitive sequence databases. (A) Six tracks show the numbers of similarity hits against reads from six Musaceae genomes as calculated by Profrep. (B) Annotation of genomic region based on our *M. acuminata* repeat annotation and Profrep analysis. (C) Annotation of repeats in the DH-Pahang genome obtained from the Banana Genome Hub.

Sequence databases specific for various types or families of repetitive elements were prepared by merging reads from clusters with the same annotations. These databases can be used for similarity-based repeat identification in assembled sequences, as implemented, for example, in our Profrep server (http://w3lamc.umbr.cas.cz/profrep/public/) [30]. Compared to approaches using databases of representative elements or consensus sequences, collections of reads gathered from clustering analysis provide better sensitivity for detecting divergent repetitive elements because they better represent their sequence variability. Examples of repeat annotation of randomly selected tracks of *M. acuminata* genome assembly [20] based on similarity hits to repeat-specific collections of sequence reads are provided in Fig. 6, S2, and S3. Identified repetitive regions (Fig. 6B) were mostly in agreement with the assembly annotation (Fig. 6C). However, it was possible to assign specific repeat types to many regions listed as unclassified repeats in the assembly annotation (e.g. most *Ty1/copia* Maximus elements in Fig. 6 or *Ty1/copia* Angela in Fig. S2). An additional benefit of our approach is the possibility to visualize abundance of corresponding repeats in other Musaceae species. For example, Angela elements present in the analyzed region of chromosome 9 are well conserved in all Musaceae species including *E. gilletii*, whereas other repeats show larger variations in their abundance (Fig. 6A). On the other hand, our approach failed to detect some of the small regions annotated as DNA transposos or unclassified repeats in the *M. acuminata* assembly (Fig. 6B–C), probably due to their very small genomic abundance, which resulted in no hits to our sequence read databases.

## Discussion

Identification of repetitive elements using graph-based clustering of sequence reads is one of the novel bioinformatic tools specifically designed to utilize the power of next generation sequencing technologies [24], [25]. This approach proved to be efficient in global repeat characterization in complex plant [24], [31], [32] and animal [33] genomes and in investigation of repeat composition of individual chromosomes [30], [34] or their compartments [35], [36]. This study employs repeat clustering methodology for comparative analysis of multiple genomes, including species representing two genera of the *Musaceae* family. It extends the previous survey of *M. acuminata* repeats [19] by analyzing more sequence data and investigating representatives of the genus covering its two taxonomic sections *Musa* and *Callimusa* [9] and three banana genomes (A, B, and T). It also complements information about repeat composition gathered from the current genome assemblies of *M. acuminata* and *M. balbisiana* [20], [26].

Although highly and moderately repeated sequences can reach up to 80–85% in plants with larger genomes [37], [38], their proportions in the investigated Musaceae species were smaller (66–71%). Such repeat proportions are slightly above repeat content estimated in species with relatively small genomes, including *Oryza* (25–66%), *Vitis vinifera* (41.4%), *Sorghum bicolor* (61%), *Malus×domestica* (67%), and *Nelumbo nucifera* (50%) [39]–[43]. The observed dominance of LTR-retrotransposons in the fraction of highly repeated sequences is a common feature of higher plant genomes where retroelements represent one of the major forces driving genome size evolution [38], [44], [45]. Although individual retroelement families varied in genome proportions in the investigated Musaceae species, there was no significant correlation of their abundance with genome size variation. Thus, genome size differences in the studied species cannot be attributed to simple amplification of particular repetitive element as seen in some other plant genomes [38], [39]. Rather, genome size was affected by joint activities of more transposable element (TE) lineages.

The observed sequence variation and quantitative differences among individual TE lineages correspond well to previous results on phylogeny of the Musaceae family. The highest similarity of repeats within the groups of *M. beccarii*/*M. textilis* and *M. acuminata*/*M. ornata* or *M. acuminata*/*M. ornata*/*M. balbisiana* are in agreement with previous results that showed close phylogenetic relationships of these species [6], [8], [46] and support the new taxonomy of Musaceae [9]. In general, sequence and quantitative differences were proportional to diversification of species of the Musaceae family (compare Fig. 1, 4, and 5). This is in agreement with previous comparative studies of repeats in genomes of the Orobanchaceae family [47] and genus *Oryza* [39]. Overall, the present and our previous studies show that repeat analysis can support the analysis of evolutionary relationships.

Clustering-based repeat analysis employed in this study provides a useful alternative to repeat quantification from genome assemblies. Comparison of our shotgun data with the published assembly of *M. acuminata* DH-Pahang [20] confirmed that repetitive sequences are under-represented in the assembly anchored into 11 *Musa* chromosomes, while repeats are overrepresented in remaining 30% sequences of un-anchored assembly (Fig. S4). However, in general, we observed good agreement between our annotation of *M. acuminata* clusters and curated TEs in the DH-Pahang assembly. Approximately 90–95% of elements annotated in the genome assembly comply with the annotation based on our clustering results. Additionally, estimates of total abundance of LTR TEs in DH-Pahang and our estimates for *M. acuminata* are very close in the cases of *Ty1/copia* and *Ty3/gypsy* (Table 2). The same *Ty1/copia* and *Ty3/gypsy* lineages were identified in genome assembly with exceptions of *Ty1/copia* Ivana, which was newly pinpointed in this study. Graph-based clustering allowed us to assign all identified LTR retrotransposons into lineages, while in genomic assembly about 46% and 24% of *Ty1/copia* and *Ty3/gypsy*, respectively, were not further classified. This is one of the benefits of graph-based clustering, as the sequence clusters contain both sequences derived from complete autonomous TEs and continuum of incomplete and mutated TEs copies, which could be difficult to detect when only similarity search against database of known repeats is used. On the other hand, the amount of DNA transposons and LINEs estimated in the present work is much lower compared with the estimates based on the genome assembly. To explain this difference, we have compared the annotation of the DH-Pahang assembly with all our *M. acuminata* sequences. The similarity search revealed that the large fraction of sequences annotated as DNA transposons and LINEs in the DH-Pahang assembly provide similarity hits to unclustered sequences or to small unannotated clusters in our data (data not shown). Consequently, it seems that we have missed these sequences in our annotation. Some differences in repeat abundance estimates can be also attributed to incompleteness of assembly and biased composition of sequences in the genome assembly.

### Conclusions

The present study provides a detailed insight into the composition and diversity of repeats in genomes representing the family Musaceae. Next generation sequencing with genome coverage greater than 10% enabled annotation and quantification of repeats that form 30–45% of the Musaceae genomes. The remaining part of the genome consists of unidentified repeats (∼30%) and low- and single-copy sequences (26–45%). We show that there is a prevalence of *Ty1/copia* elements in all *Musa* genomes, with a majority of *Ty1/copia* elements being Maximus/SIRE and Angela. Most of the elements in *Ty3/gypsy* family belong to the lineage of chromoviruses. The present study revealed significant divergence in repeat composition between the species of Musa, and the extent of repeat divergence was related to the estimated divergence dates of the species in the *Musaceae* family. We also demonstrated that database of repeats derived from graph based clustering is well suited for annotation of genome assemblies and can complement other repeat annotation methods.

## Materials and Methods

### Plant material, DNA isolation and sequencing

*In vitro* rooted plants of most of the *Musa* and *Ensete* species used in this study were obtained from the International Transit Centre (ITC, Katholieke Universiteit Leuven, Belgium). The clone ‘Pisang Klutuk Wulung’ of *M. balbisiana* was obtained from CIRAD (Guadeloupe) as rooted plants. Plants were transferred to soil and maintained in a greenhouse.

Genomic DNA of *M. acuminata* ‘Calcutta4’ (ITC 0249), *M. balbisiana* ‘Pisang Klutuk Wulung’, *M. ornata* (ITC 0637), *M. textilis* (ITC 0539) and *M. beccarii* (ITC 1070) was prepared from nuclei isolated from healthy young leaf tissue according to [48]. Intact nuclei of *E. gilletii* (ITC 1389) were isolated by flow cytometric sorting following the protocol of [49]. Isolated nuclei were incubated with 40 mM EDTA, 0.2% SDS and 0.25 µg/µl proteinase K for 5 hrs at 37°C, and nuclear DNA was purified by phenol/chloroform extraction.

Roche/454 shotgun sequencing libraries were prepared by the GS Titanium library preparation kit (454 Life Sciences, a Roche company, Branford, USA). The single-stranded libraries were quantified by a qPCR assay and processed utilizing the GS Titanium SV/LV emPCR and XLR70 sequencing kits according to the manufacturer's instructions (Roche Diagnostics). Sequencing was performed on a half 70×75 picotiter plate for each *Musa* cultivar [8]. Sequence reads were divided into clusters using a graph-based method according to [24] with the difference in that the reads were trimmed to 200 nucleotides and reads from all species were clustered together. Computational tools used for clustering step are available at the public server (www.repeatexplorer.org) [25].

### Repeat cluster annotation and repeat identification

Several resources were used to manually annotate clusters. Reads from clusters were scanned for similarity to a database of plant repetitive elements with RepeatMasker [50] using databases that were improved by adding specific sequences derived from the banana genome based on our previous work [19]. Blastx and blastn [51] were used for similarity search against public databases and also against our database of protein domains derived from plant mobile genetic elements. Clusters represented as graphs were also analyzed using SeqGrapheR program (http://cran.rproject.org/web/packages/SeqGrapheR/index.html).

### Musaceae phylogenetic tree construction

Internal transcribed spacers (ITS) data obtained by [19] was used to construct a BioNJ tree based on the Jukes-Cantor model in the SeaView v4.2.3 program [52]. Phylogenetic trees were drawn and edited using the FigTree (http://tree.bio.ed.ac.uk/software/figtree/) program.

### Construction of phylogenetic tree for RT domains

Reads with similarity to the *Ty3/gypsy* reverse-transcriptase domain were trimmed and aligned using MAFFT software [53], and the maximum-likelihood phylogenetic tree was estimated using the FastTree program [54]. The resulting alignment and tree are provided in Files S1 and S2.

### Southern blots

Genomic DNA of 15 selected Musaceae representatives was prepared from nuclei isolated from healthy young leaf tissue. Aliquots of genomic DNA samples corresponding to16×10^6^ of nuclear genomes were digested using *Dra*I, *Eco*RV, *Rsa*I or *Msp*I restriction enzymes, size-fractionated by 1.2% agarose gel electrophoresis, and transferred onto Hybond N+ nylon membranes (Amersham Biosciences, Bath, UK). Biotin-labeled oligomers (file S3) were used as probes. The Southern hybridization was done at 68°C overnight followed by stringent washes (stringency 90%). Signals were detected using the BrightStar BioDetect kit according to the manufacturer's instructions (Ambion, Austin, USA), incubated with chemiluminescent substrate (CDP-Star, Amersham Biosciences), and exposed to X-ray film.

### Resources

Sequences were deposited in the Sequence Read Archive under accession ERX047938–ERX047944.

Profrep with Musaceae repeat database is available at http://w3lamc.umbr.cas.cz/profrep/public.

## Supporting Information

Figure S1 **Clustering of sequence reads.** Top bar plot show the results of clustering based on the hierarchical agglomeration algorithm of all sequence reads. Each bar represents one cluster; the height and width of the bars correspond to the number of reads in the clusters. The Y-axis shows the percentage of the reads in the clusters and the X-axis shows their cumulative content. Bars are colored according to the major types of identified repeats. Only first 106 largest clusters is shown. Six lower bar plots show the representation of reads in the clusters by individual species.
(PNG)

Figures S2 **DH-Pahang genomic sequences together with repeat annotation were obtained from the Banana Genome Hub (** http://banana-genome.cirad.fr/ **) and analyzed using the Profrep tool against our Musaceae sequence database.** (A) Six tracks show the number of similarity hits against reads from six Musaceae genomes as calculated by Profrep. (B) Annotation of genomic region based on our *M. acuminata* repeat annotation and Profrep analysis. (C) Annotation of repeats in the DH-Pahang genome obtained from the Banana Genome Hub.
(PNG)

Figure S3 **DH-Pahang genomic sequences together with repeat annotation were obtained from the Banana Genome Hub (** http://banana-genome.cirad.fr/ **) and analyzed using the Profrep tool against our Musaceae sequence database.** (A) Six tracks show the number of similarity hits against reads from six Musaceae genomes as calculated by Profrep. (B) Annotation of genomic region based on our *M. acuminata* repeat annotation and Profrep analysis. (C) Annotation of repeats in the DH-Pahang genome obtained from the Banana Genome Hub.
(PNG)

Figure S4 **Comparison of repetitive content in shotgun sequencing data and genome assemblies of** ***M. acuminata*** **and** ***M. balbisiana*** **.** Each dot represents a sequence read. The X and Y axes show the normalized number of similarity hits detected in shogun data and assembly, respectively.
(PNG)

File S1 **DNA alignment of reverse transcriptase sequences derived from the** ***Ty3/gypsy*** **CRM element from cluster CL18.**
(FAS)

File S2 **Phylogenetic tree estimated by FastTree program from sequences derived from the** ***Ty3/gypsy*** **CRM element from cluster CL18.**
(TREE)

File S3 **Sequences of oligonucleotide probes used in Southern blot hybridization.**
(TXT)

## References

[1] Cheesman EE (2008) Classification of the Bananas: The Genus *Musa* L.

[2] Risterucci A, Hippolyte I, Perrier X (2009) Development and assessment of Diversity Arrays Technology for high-throughput DNA analyses in *Musa* . Theor Appl Genet 119: 1093–1103. 19693484 10.1007/s00122-009-1111-5

[3] Loh JP, Kiew R, Set O, Gan LH, Gan YY (2000) Amplified fragment length polymorphism fingerprinting of 16 banana cultivars (*Musa* cvs.). Mol Phylogenet Evol 17: 360–366. 11133190 10.1006/mpev.2000.0848

[4] Wong C (2001) Genetic diversity of the wild banana *Musa acuminata Colla* in Malaysia as evidenced by AFLP. Ann Bot 88: 1017–1025.

[5] Ruangsuttapha S, Eimert K, Schröder M-B, Silayoi B, Denduangboripant J, et al (2007) Molecular phylogeny of banana cultivars from Thailand based on HAT-RAPD markers. Genet Resour Crop Evol 54: 1565–1572.

[6] Li L-F, Häkkinen M, Yuan Y-M, Hao G, Ge X-J (2010) Molecular phylogeny and systematics of the banana family (Musaceae) inferred from multiple nuclear and chloroplast DNA fragments, with a special reference to the genus *Musa* . Mol Phylogenet Evol 57: 1–10. 20601004 10.1016/j.ympev.2010.06.021

[7] Christelová P, Valárik M, Hřibová E, Van den Houwe I, Channelière S, et al (2011) A platform for efficient genotyping in *Musa* using microsatellite markers. AoB Plants 2011: plr024. 22476494 10.1093/aobpla/plr024 PMC3185971

[8] Hřibová E, Čížková J, Christelová P, Taudien S, de Langhe E, et al (2011) The ITS1-5.8S-ITS2 sequence region in the Musaceae: structure, diversity and use in molecular phylogeny. PLoS One 6: e17863. 21445344 10.1371/journal.pone.0017863 PMC3062550

[9] Häkkinen M (2013) Reappraisal of sectional taxonomy in *Musa* (Musaceae). Taxon 62: 809–813.

[10] Simmonds NW, Shepherd K (1955) The taxonomy and origins of the cultivated bananas. J Linn Soc London, Bot 55: 302–312.

[11] Heslop-Harrison JS, Schwarzacher T (2007) Domestication, genomics and the future for banana. Ann Bot 100: 1073–1084. 17766312 10.1093/aob/mcm191 PMC2759213

[12] Simmonds NW (1956) Botanical results of the banana collecting expedition 1954–5. Kew Bull 3: 463–489.

[13] Cheesman EE (1950) Classification of the bananas. III: Critical notes on species. Kew Bull 5: 27–28.

[14] Jarret RL, Gawel N, Whittemore A, Sharrock S (1992) RFLP-based phylogeny of *Musa* species in Papua New Guinea. Theor Appl Genet 84: 579–584. 24201344 10.1007/BF00224155

[15] Carreel F (1994) Etude de la diversité des bananiers (genre *Musa*) à l'aide des marquers RFLP Institut National Agronomique, Paris-Grignon, France.

[16] Doležel J, Doleželová M, Novák FJ (1994) Flow cytometric estimation of nuclear DNA amount in diploid bananas (*Musa acuminata* and *M. balbisiana*). Biol Plant 36: 351–357.

[17] Bartoš J, Alkhimova O, Doleželová M, De Langhe E, Doležel J (2005) Nuclear genome size and genomic distribution of ribosomal DNA in *Musa* and *Ensete* (Musaceae): taxonomic implications. Cytogenet Genome Res 109: 50–57. 15753558 10.1159/000082381

[18] Hřibová E, Doleželová M, Town CD, Macas J, Doležel J (2007) Isolation and characterization of the highly repeated fraction of the banana genome. Cytogenet Genome Res 119: 268–274. 18253041 10.1159/000112073

[19] Hřibová E, Neumann P, Matsumoto T, Roux N, Macas J, et al (2010) Repetitive part of the banana (*Musa acuminata*) genome investigated by low-depth 454 sequencing. BMC Plant Biol 10: 204. 20846365 10.1186/1471-2229-10-204 PMC2956553

[20] D'Hont A, Denoeud F, Aury J-M, Baurens F-C, Carreel F, et al (2012) The banana (*Musa acuminata*) genome and the evolution of monocotyledonous plants. Nature 488: 213–217. 22801500 10.1038/nature11241

[21] Ellis TH, Poyser SJ, Knox MR, Vershinin A V, Ambrose MJ (1998) Polymorphism of insertion sites of *Ty1-copia* class retrotransposons and its use for linkage and diversity analysis in pea. Mol Gen Genet 260: 9–19. 9829823 10.1007/pl00008630

[22] Kalendar R, Grob T, Regina M, Suoniemi A, Schulman A (1999) IRAP and REMAP: two new retrotransposon-based DNA fingerprinting techniques. Theor Appl Genet 98: 704–711.

[23] Purugganan MD, Wessler SR (1995) Transposon signatures: species-specific molecular markers that utilize a class of multiple-copy nuclear DNA. Mol Ecol 4: 265–270. 7735530 10.1111/j.1365-294x.1995.tb00218.x

[24] Novák P, Neumann P, Macas J (2010) Graph-based clustering and characterization of repetitive sequences in next-generation sequencing data. BMC Bioinformatics 11: 378. 20633259 10.1186/1471-2105-11-378 PMC2912890

[25] Novák P, Neumann P, Pech J, Steinhaisl J, Macas J (2013) RepeatExplorer: a Galaxy-based web server for genome-wide characterization of eukaryotic repetitive elements from next-generation sequence reads. Bioinformatics 29: 792–793. 23376349 10.1093/bioinformatics/btt054

[26] Davey MW, Gudimella R, Harikrishna JA, Sin LW, Khalid N, et al (2013) “A draft *Musa balbisiana* genome sequence for molecular genetics in polyploid, inter- and intra-specific *Musa* hybrids.”. BMC Genomics 14: 683. 24094114 10.1186/1471-2164-14-683 PMC3852598

[27] Čížková J, Hřibová E, Humplíková L, Christelová P, Suchánková P, et al (2013) Molecular analysis and genomic organization of major DNA satellites in banana (*Musa* spp.). PLoS One 8: e54808. 23372772 10.1371/journal.pone.0054808 PMC3553004

[28] Lander ES, Waterman MS (1988) Genomic mapping by fingerprinting random clones: a mathematical analysis. Genomics 2: 231–239. 3294162 10.1016/0888-7543(88)90007-9

[29] Neumann P, Navrátilová A, Koblížková A, Kejnovský E, Hřibová E, et al (2011) Plant centromeric retrotransposons: a structural and cytogenetic perspective. Mob DNA 2: 4. 21371312 10.1186/1759-8753-2-4 PMC3059260

[30] Macas J, Kejnovský E, Neumann P, Novák P, Koblížková A, et al (2011) Next generation sequencing-based analysis of repetitive DNA in the model dioecious plant *Silene latifolia* . PLoS One 6: e27335. 22096552 10.1371/journal.pone.0027335 PMC3212565

[31] Renny-Byfield S, Chester M, Kovařík A, Le Comber SC, Grandbastien M-A, et al (2011) Next generation sequencing reveals genome downsizing in allotetraploid *Nicotiana tabacum*, predominantly through the elimination of paternally derived repetitive DNAs. Mol Biol Evol 28: 2843–2854. 21512105 10.1093/molbev/msr112

[32] Heckmann S, Macas J, Kumke K, Fuchs J, Schubert V, et al (2013) The holocentric species *Luzula elegans* shows interplay between centromere and large-scale genome organization. Plant J 73: 555–565. 23078243 10.1111/tpj.12054

[33] Pagan HJT, Macas J, Novák P, McCulloch ES, Stevens RD, et al (2012) Survey sequencing reveals elevated DNA transposon activity, novel elements, and variation in repetitive landscapes among vesper bats. Genome Biol Evol 4: 575–585. 22491057 10.1093/gbe/evs038 PMC3342881

[34] Martis MM, Klemme S, Banaei-Moghaddam AM, Blattner FR, Macas J, et al (2012) Selfish supernumerary chromosome reveals its origin as a mosaic of host genome and organellar sequences. Proc Natl Acad Sci U S A 109: 13343–13346. 22847450 10.1073/pnas.1204237109 PMC3421217

[35] Neumann P, Navrátilová A, Schroeder-Reiter E, Koblížková A, Steinbauerová V, et al (2012) Stretching the rules: monocentric chromosomes with multiple centromere domains. PLoS Genet 8: e1002777. 22737088 10.1371/journal.pgen.1002777 PMC3380829

[36] Gong Z, Wu Y, Koblížková A, Torres GA, Wang K, et al (2012) Repeatless and repeat-based centromeres in potato: implications for centromere evolution. Plant Cell 24: 3559–3574. 22968715 10.1105/tpc.112.100511 PMC3480287

[37] Staton SE, Bakken BH, Blackman BK, Chapman MA, Kane NC, et al (2012) The sunflower (*Helianthus annuus* L.) genome reflects a recent history of biased accumulation of transposable elements. Plant J 72: 142–153. 22691070 10.1111/j.1365-313X.2012.05072.x

[38] Tenaillon MI, Hufford MB, Gaut BS, Ross-Ibarra J (2011) Genome size and transposable element content as determined by high-throughput sequencing in maize and *Zea luxurians* . Genome Biol Evol 3: 219–229. 21296765 10.1093/gbe/evr008 PMC3068001

[39] Zuccolo A, Sebastian A, Talag J, Yu Y, Kim H, et al (2007) Transposable element distribution, abundance and role in genome size variation in the genus *Oryza* . BMC Evol Biol 7: 152. 17727727 10.1186/1471-2148-7-152 PMC2041954

[40] Jaillon O, Aury J-M, Noel B, Policriti A, Clepet C, et al (2007) The grapevine genome sequence suggests ancestral hexaploidization in major angiosperm phyla. Nature 449: 463–467. 17721507 10.1038/nature06148

[41] Velasco R, Zharkikh A, Affourtit J, Dhingra A, Cestaro A, et al (2010) The genome of the domesticated apple (*Malus*×*domestica* Borkh.). Nat Genet 42: 833–839. 20802477 10.1038/ng.654

[42] Paterson AH, Bowers JE, Bruggmann R, Dubchak I, Grimwood J, et al (2009) The *Sorghum bicolor* genome and the diversification of grasses. Nature 457: 551–556. 19189423 10.1038/nature07723

[43] Wang Y, Fan G, Liu Y, Sun F, Shi C, et al (2013) The sacred lotus genome provides insights into the evolution of flowering plants. Plant J 76: 557–567. 23952714 10.1111/tpj.12313

[44] Neumann P, Koblížková A, Navrátilová A, Macas J (2006) Significant expansion of *Vicia pannonica* genome size mediated by amplification of a single type of giant retroelement. Genetics 173: 1047–1056. 16585134 10.1534/genetics.106.056259 PMC1526492

[45] Hawkins JS, Kim H, Nason JD, Wing RA, Wendel JF (2006) Differential lineage-specific amplification of transposable elements is responsible for genome size variation in *Gossypium* . Genome Res 16: 1252–1261. 16954538 10.1101/gr.5282906 PMC1581434

[46] Christelová P, Valárik M, Hřibová E, De Langhe E, Doležel J (2011) A multi gene sequence-based phylogeny of the Musaceae (banana) family. BMC Evol Biol 11: 103. 21496296 10.1186/1471-2148-11-103 PMC3102628

[47] Piednoël M, Aberer AJ, Schneeweiss GM, Macas J, Novák P, et al (2012) Next-generation sequencing reveals the impact of repetitive DNA across phylogenetically closely related genomes of Orobanchaceae. Mol Biol Evol 29: 3601–3611. 22723303 10.1093/molbev/mss168 PMC3859920

[48] Zhang H-B, Zhao X, Ding X, Paterson AH, Wing RA (1995) Preparation of megabase-size DNA from plant nuclei. Plant J 7: 175–184.

[49] Šafář J, Bartoš J, Janda J, Bellec A, Kubaláková M, et al (2004) Dissecting large and complex genomes: flow sorting and BAC cloning of individual chromosomes from bread wheat. Plant J 39: 960–968. 15341637 10.1111/j.1365-313X.2004.02179.x

[50] Smit A, Hubley R, Green P (1996) RepeatMasker Open-3.0.

[51] Altschul SF, Gish W, Miller W, Myers EW, Lipman DJ (1990) Basic local alignment search tool. J Mol Biol 215: 403–410. 2231712 10.1016/S0022-2836(05)80360-2

[52] Galtier N, Gouy M, Gautier C (1996) SEAVIEW and PHYLO_WIN: two graphic tools for sequence alignment and molecular phylogeny. Bioinformatics 12: 543–548. 10.1093/bioinformatics/12.6.543 9021275

[53] Katoh K, Standley DM (2013) MAFFT multiple sequence alignment software version 7: improvements in performance and usability. Mol Biol Evol 30: 772–780. 23329690 10.1093/molbev/mst010 PMC3603318

[54] Price MN, Dehal PS, Arkin AP (2009) FastTree: computing large minimum evolution trees with profiles instead of a distance matrix. Mol Biol Evol 26: 1641–1650. 19377059 10.1093/molbev/msp077 PMC2693737

[55] Fruchterman TMJ, Reingold EM (1991) Graph drawing by force-directed placement. Softw Pract Exp 21: 1129–1164.

