# Supplementary figures and images for: Genome-Wide Analysis of Repeat Diversity across the Family Musaceae
Source: PLoS One. 2014 Jun 16;9(6):e98918. doi: 10.1371/journal.pone.0098918 (PMC4059648; doi:10.1371/journal.pone.0098918)

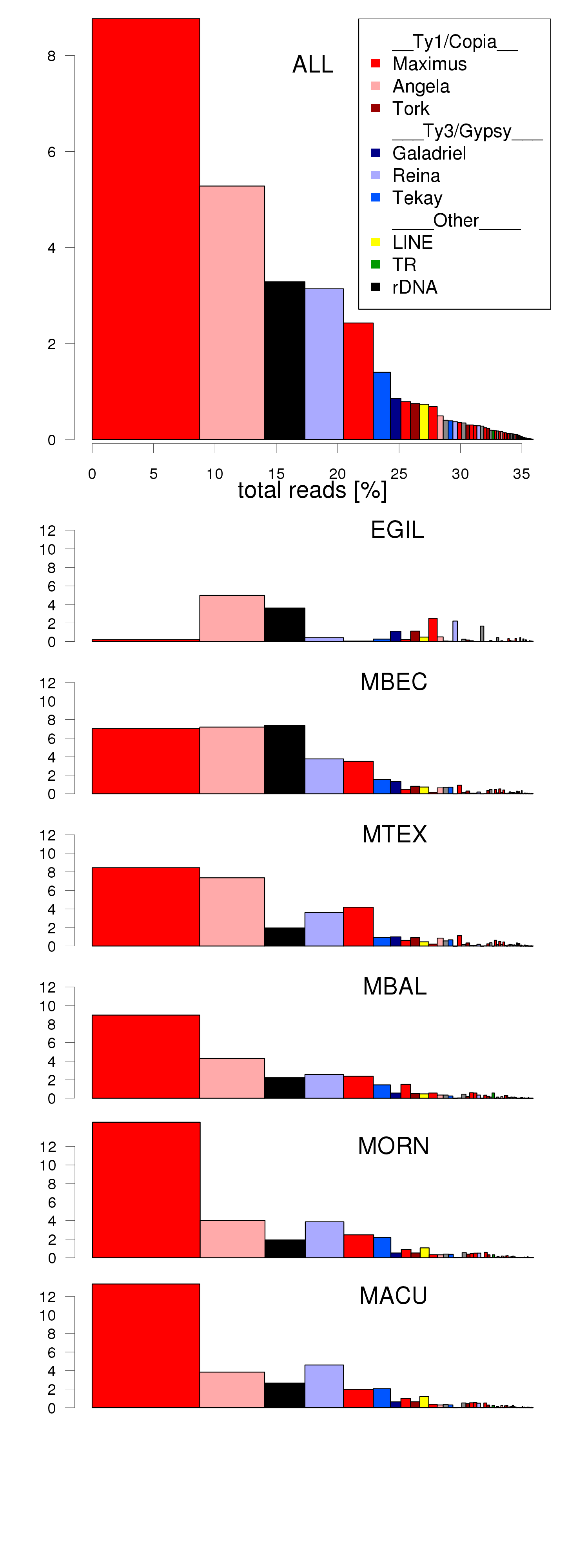

Supplement: Figure S1 — Clustering of sequence reads. Top bar plot show the results of clustering based on the hierarchical agglomeration algorithm of all sequence reads. Each bar represents one cluster; the height and width of the bars correspond to the number of reads in the clusters. The Y-axis shows the percentage of the reads in the clusters and the X-axis shows their cumulative content. Bars are colored according to the major types of identified repeats. Only first 106 largest clusters is shown. Six lower bar plots show the representation of reads in the clusters by individual species. (PNG) [file pone.0098918.s001.png]

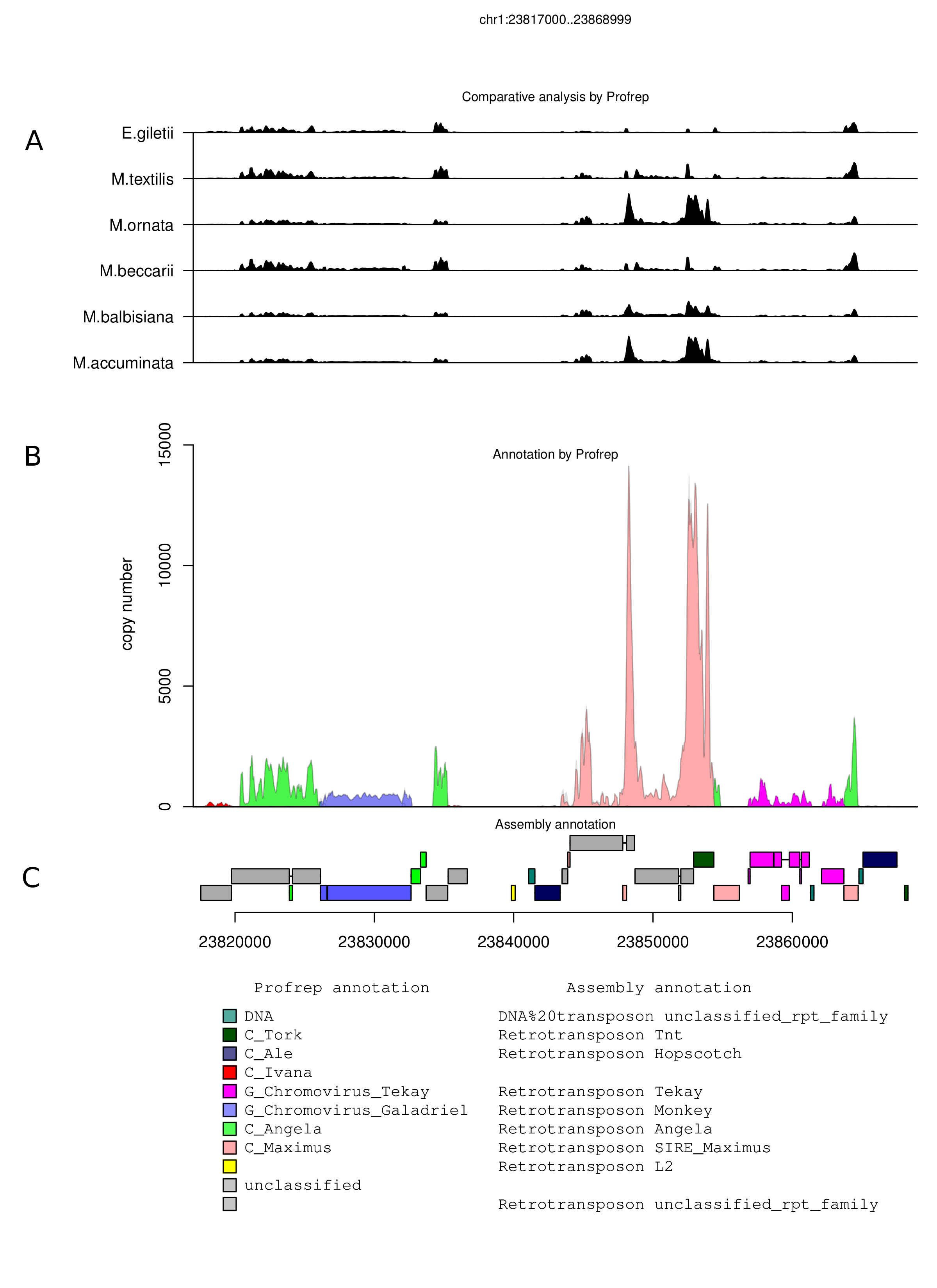

Supplement: Figures S2 — DH-Pahang genomic sequences together with repeat annotation were obtained from the Banana Genome Hub ( http://banana-genome.cirad.fr/ ) and analyzed using the Profrep tool against our Musaceae sequence database. (A) Six tracks show the number of similarity hits against reads from six Musaceae genomes as calculated by Profrep. (B) Annotation of genomic region based on our M. acuminata repeat annotation and Profrep analysis. (C) Annotation of repeats in the DH-Pahang genome obtained from the Banana Genome Hub. (PNG) [file pone.0098918.s002.png]

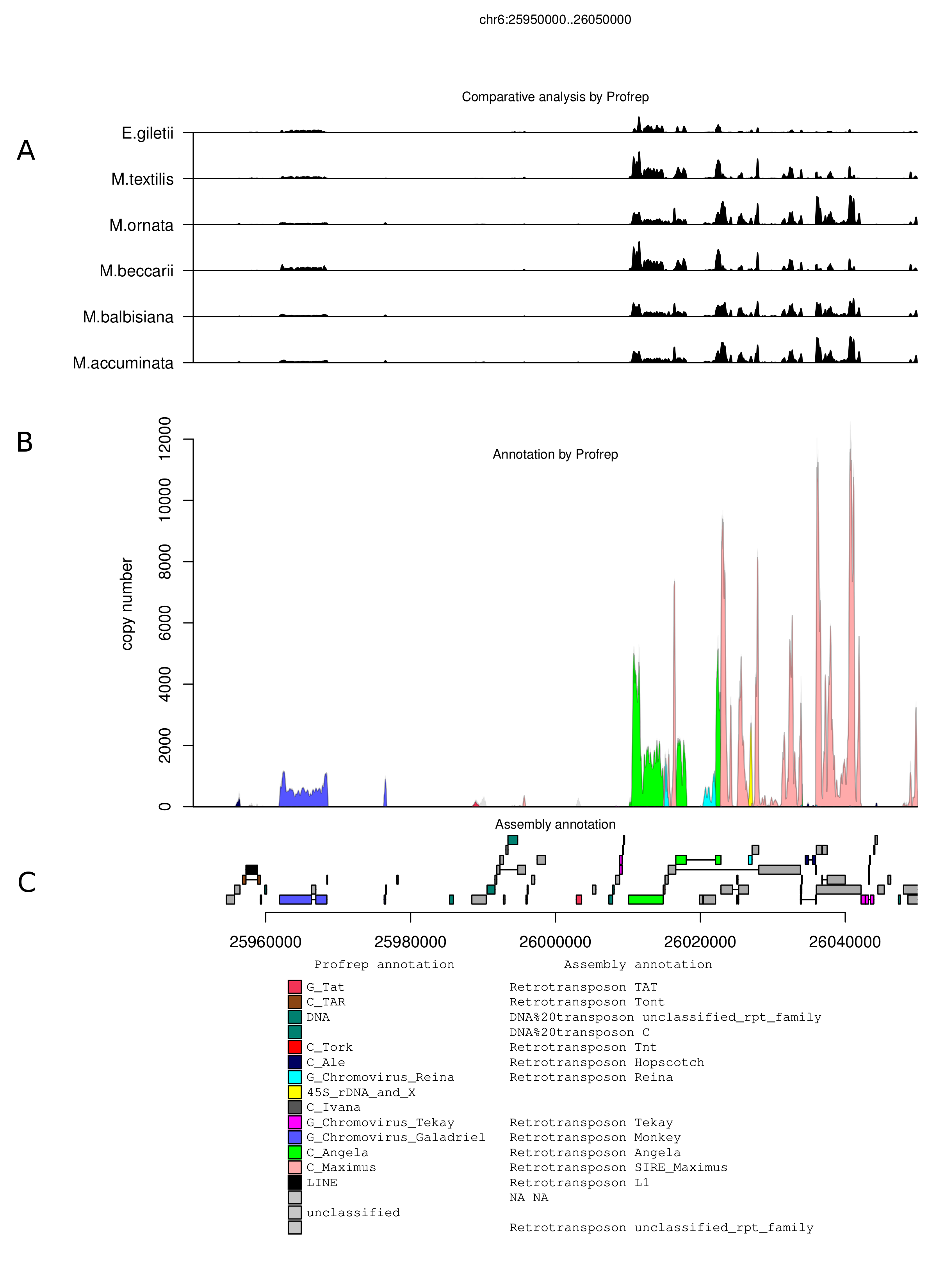

Supplement: Figure S3 — DH-Pahang genomic sequences together with repeat annotation were obtained from the Banana Genome Hub ( http://banana-genome.cirad.fr/ ) and analyzed using the Profrep tool against our Musaceae sequence database. (A) Six tracks show the number of similarity hits against reads from six Musaceae genomes as calculated by Profrep. (B) Annotation of genomic region based on our M. acuminata repeat annotation and Profrep analysis. (C) Annotation of repeats in the DH-Pahang genome obtained from the Banana Genome Hub. (PNG) [file pone.0098918.s003.png]

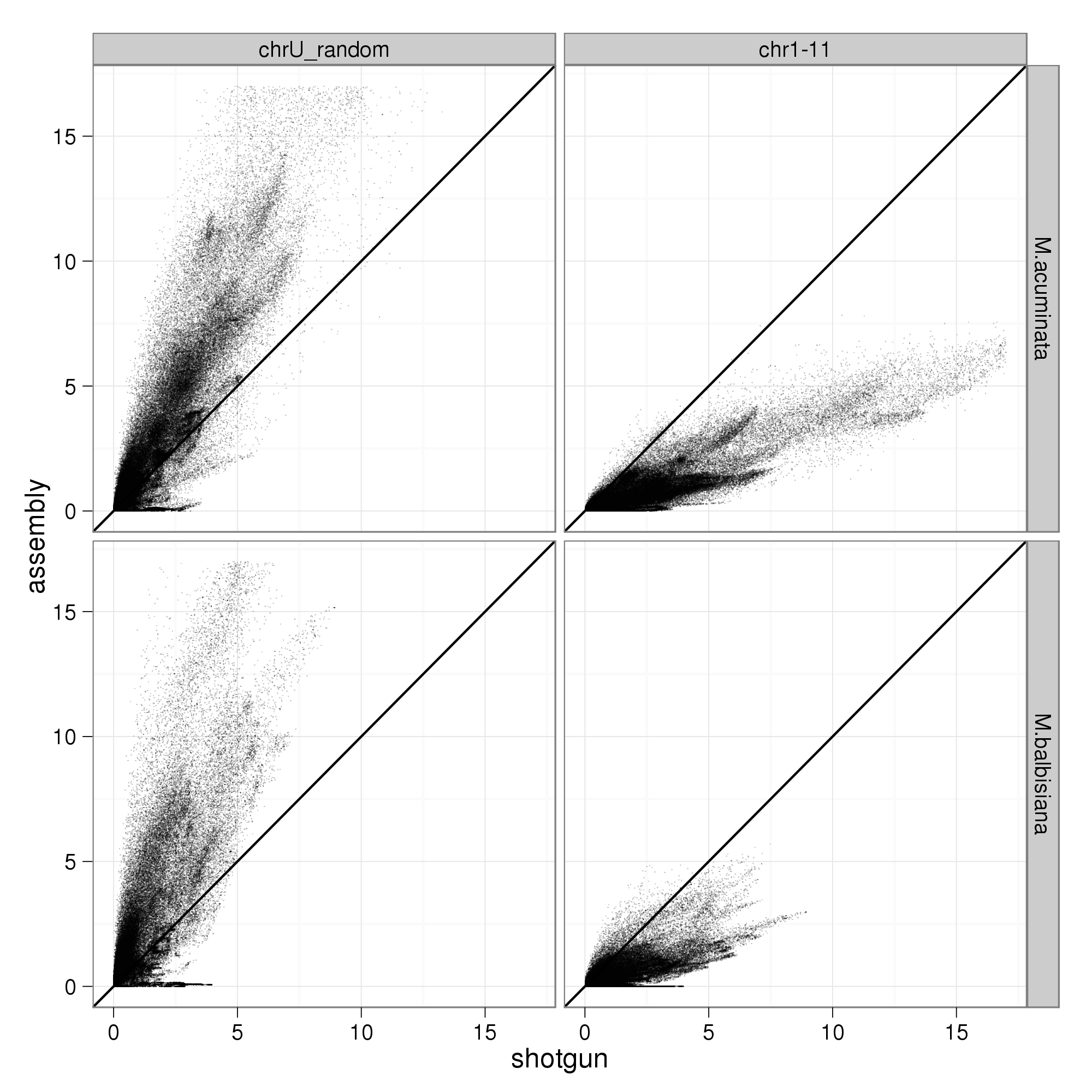

Supplement: Figure S4 — Comparison of repetitive content in shotgun sequencing data and genome assemblies of M. acuminata and M. balbisiana . Each dot represents a sequence read. The X and Y axes show the normalized number of similarity hits detected in shogun data and assembly, respectively. (PNG) [file pone.0098918.s004.png]
